# Simple Recommendations for Improving Efficiency in Generating Genome-Edited Mice

**DOI:** 10.32607/actanaturae.10937

**Published:** 2020

**Authors:** O. A. Averina, M. Y. Vysokikh, O. A. Permyakov, P. V. Sergiev

**Affiliations:** Institute of functional genomics, Lomonosov Moscow State University, Moscow, 119991 Russia; Belozersky Institute of Physico-Chemical Biology, Lomonosov Moscow State University, Moscow, 119991 Russia; Department of Chemistry, Lomonosov Moscow State University, Moscow, 119991 Russia

**Keywords:** genome editing, transgenic mice, superovulation, mouse zygote donors, mouse foster mothers

## Abstract

The generation of transgenic model organisms (primarily mice) is an integral part of modern fundamental and applied research. Simple techniques based on the biology of these laboratory rodents can often increase efficiency when generating genome-edited mouse strains. In this study, we share our three years of experience in the optimization of mouse genome editing based on microinjection of CRISPR/Cas9 components into ca. 10,000 zygotes. We tested a number of techniques meant to improve efficiency in generating knockout mice, such as optimization of the superovulation method and choosing the optimal mouse strains to be used as zygote donors and foster mothers. The presented results might be useful to laboratories aiming to quickly and efficiently create new mouse strains with tailored genome editing.

## INTRODUCTION

**Fig. 1:**
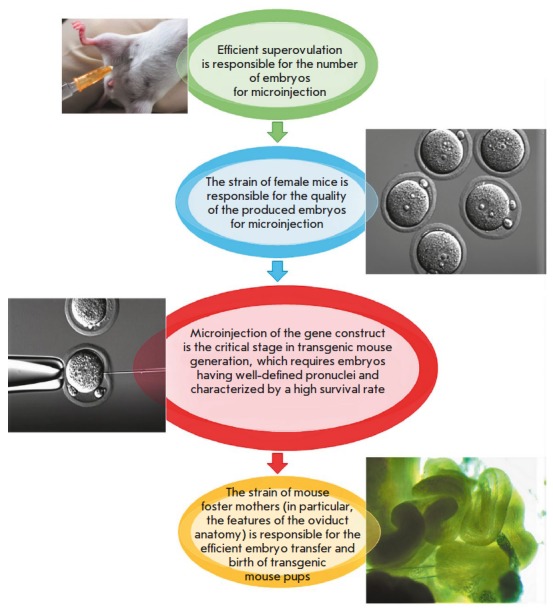
Scheme for generating genome-edited mice and troubleshooting at each stage

The mouse genome editing technology was elaborated in the 1980s [1-5] and aims to study gene functions and the genetic mechanisms underlying the emergence of human diseases, as well as to develop methods for their treatment [6, 7]. This technology has a significant impact on such interrelated disciplines as veterinary and agriculture [8, 9]. The first protocols for generating genome-edited mice were published over 30 years ago. Today, the technology continues to be mastered; the main efforts in the research focus on the development of novel molecular tools for genome editing [10-13]. Meanwhile, the technical aspects of producing mice are very important in the generation of genome-edited mice. Regardless of the genome-editing tool being used, the protocol for generating genome-edited mice comprises several stages. The first stage consists in subjecting female mice used as zygote donors to superovulation and mating them with males. The second stage consists in zygote isolation and microinjection of the components of the system for genome editing. The third stage involves the implantation of microinjected zygotes into the oviduct of a pseudopregnant recipient female mouse, pregnancy, and nursing of mouse pups. Each stage in this process needs to be optimized to achieve maximum efficiency (*Fig. 1*). The researcher’s objective was to achieve the optimal conditions for producing the maximum possible number of zygotes that can be used for microinjections and subsequent efficient embryo transfer. The maximum number of viable mouse pups subsequently reaching reproductive age needs to be born.

The reported data were collected during a threeyear period. More than 10,000 zygotes were isolated from ca. 850 mice. The zygotes were transferred to more than 300 mouse foster mothers, which gave birth to more than 380 genome-edited pups. Thirtyfour mouse strains with 16 edited genes were selected (*Table*).

**Table 1:** Significant optimization variables for the technology for generating genome-edited mice

Efficiency of producing zygotes suitable for a microinjection								
Mouse strain	Type of hormonal treatment	Number of female mice subjected to superovulation and mated with male mice	Number of fertilized mice	Median percentage of the mated mice	Number of isolated zygotes	Median number of zygotes per mouse	Number of zygotes that survived microinjection	Median percentage of zygotes that survived microinjection with respect to the total number of isolated zygotes
(C57Bl/6 × CBA) F1 hybrid	eCG & hCG	2007	619	0.40	5124	8.39	2215	0.43
(C57Bl/6 × CBA) F1 hybrid	Inhibin antiserum + eCG & hCG	166	105	0.67	3499	33.20	1191	0.33
Inbred CBA strain	eCG & hCG	540	92	0.15	721	5.11	386	0.50
Inbred FVB strain	Inhibin antiserum + eCG & hCG	105	64	0.60	1449	22.83	696	0.41
Efficiency of producing offspring from mouse foster mothers after transferring zygotes subjected to microinjection								
Mouse strain		Number of transferred microinjected embryos		Number of mouse pups born		Median percentage of mouse pups that were born with respect to the number of transferred embryos		
(C57Bl/6 × CBA) F1 hybrid		1361		145		0.053		
Outbred CD1 strain		1532		154		0.040		

## EXPERIMENTAL

**Study object**

Laboratory mice procured from the Federal Research Center Institute of Cytology and Genetics, Siberian Branch, Russian Academy of Sciences (ICG SB RAS) (Novosibirsk, Russia) were used in this study. All manipulations were conducted in compliance with the protocol approved by the Local Bioethics Commission of the Research Center “Institute of Mitoengineering of Moscow State University” LLC, (Moscow, Russia) (http://www.vec-msu.ru/), Commission decision No. 67 dated April 28, 2015. The following animals were used as zygote donors: 713 female (C57Bl/6 × CBA) F1 hybrid mice (F1), 92 female inbred CBA mice, and 55 female inbred FVB mice. The zygote donors (46 F1 and 46 FVB female mice) were mated with ten male F1 hybrid mice and ten male FVB mice. Ten male F1 hybrid mice and ten outbred CD1 mice were vasectomized and mated with zygote donor females. The estrous cycle in ten female CBA and F1 hybrid mice was analyzed.

**Housing conditions of the laboratory mice**

The animals were kept in individually ventilated cages (IVC system, TECNIPLAST S.p.A., Italy), five animals per cage, with unrestricted access to food (granulated autoclaved feed manufactured by Sniff Spezialdiaten GmbH, Germany) and water purified by reverse osmosis, in an environment free of specific pathogens, under a 12 : 12 h light/dark cycle (light was turned on at 9 a.m.). The air change coefficient in the room was ≥ 15 air changes per h; air temperature was 20–24°C; and humidity was 30–0%. Lignocel wood chips (JRS, Germany) were used as bedding. The animals were exposed only to sterile materials.

**Obtaining zygotes for microinjections**

In order to obtain zygotes, the mice were subjected to superovulation via intraperitoneal administration of hormones according to two protocols:

1) 200 μl (8 MU) of equine chorionic gonadotropin (eCG) (FollimagR, ZAO Mosagrogen, Russia) injected between 10 a.m. and 12 p.m., followed by an injection of 200 μl (8 MU) of human chorionic gonadotropin (hCG) (ChorulonR, MSD Animal Health, Merck, Netherlands) after 48 h;

2) 100–140 μl of inhibin antiserum + eCG (CARD HyperOvaR, Cosmobio LTD, Japan, Patent JP 5,927,588) at 5 p.m. followed by an injection of 200 μl of human chorionic gonadotropin (hCG) (ChorulonR, MSD Animal Health, Merck, Netherlands) after 48 h, at 3 p.m.

After administering hCG, the female mice were mated with males of the respective strain. The fertilization success was evaluated the following day based on the presence of vaginal plugs [14]. The ovary and oviduct were removed, and zygotes were subsequently isolated according to the protocol proposed by Cho (2009) [6].

**Embryo transfer to mouse foster mothers**

Pseudopregnancy was induced in mouse foster mothers prior to surgery to ensure successful embryo implantation. The female mice were mated with vasectomized males and tested for the presence of vaginal plugs (an indicator of pseudopregnancy) on the day of surgery [14, 15]. Embryos were transferred into the oviductal infundibulum according to the protocol proposed by Cho (2009) [6].

**Analysis of the estrous cycle**

In order to evaluate the estrous cycle regularity in the mouse zygote donors, vaginal smears were collected at the same time of the day during 14 days using the procedure described by Ekambaram (2017) [16]. Estrous cycle stages were determined according to the cell composition of vaginal smears [17-19].

**Statistical data analysis**

Statistical data analysis was performed using the nonparametric Mann–Whitney U test. The results are presented as (median; 0.25–0.75 quantile range).

## RESULTS AND DISCUSSION

This study focused on approaches to optimize zygote production to ensure efficient microinjection and minimize loss during subsequent manipulations. When generating transgenic mice, the priority is to produce as many high-quality zygotes from a single mouse for microinjection as possible. We analyzed the efficiency of various superovulation protocols for mouse zygote donors. In order to increase the chances for embryo survival after implantation of the gene construct, we selected a mouse strain whose females produced zygotes that are more resistant to the penetration of a microinjection needle and more suitable to pronuclear transfer thanks to their structure. The next critical stage involved transfer of the microinjected zygotes to a mouse foster mother and their intrauterine and postnatal development. In this connection, we chose a mouse strain whose females made the best foster mothers in terms of such criteria as fertility and good maternal behavior. Another factor taken into account when choosing the mouse strain was the efficiency of microsurgical embryo transfer surgery.

**Choosing the superovulation method**

The number of ovulated oocytes is an important factor that affects efficiency in generating genome-edited mice. The efficiency of oocyte release is enhanced using the superovulation methods, which artificially stimulate folliculogenesis and cause hormone-induced ovulation. In mice, superovulation has conventionally been induced by using a combination of eCG and hCG hormones [20, 21]. The efficiency of this superovulation scheme depends not only on the mouse strain, but also on the quality of hormonal agents, which differ significantly for different manufacturers.

Over the past years, it has been shown that injection of the inhibin antiserum stimulates superovulation [22]. Inhibin is a protein hormone which affects pituitary cells and inhibits the secretion of the folliclestimulating hormone [23]. Inactivation of inhibin by the antiserum promotes follicle maturation [24]. The superovulation scheme has recently been modernized: now, the first stage of the stimulation involves simultaneous injection of a eCG-containing serum and anti-inhibin antibodies [25].

**Fig. 2:**
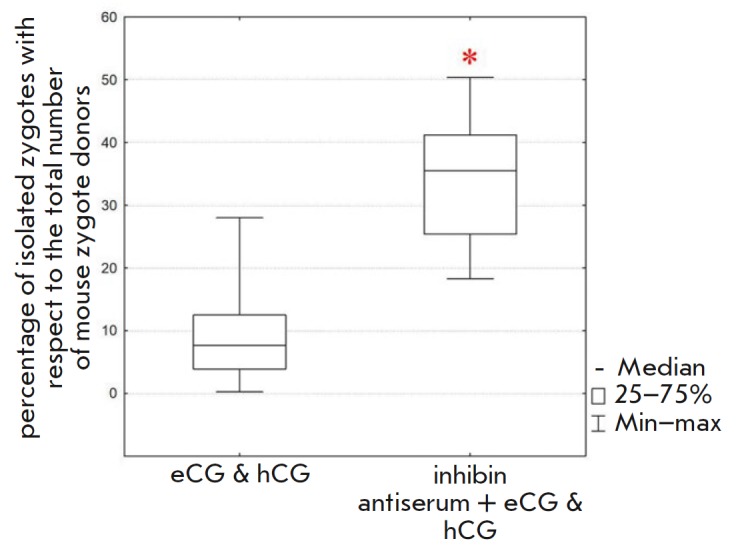
The influence of the superovulation method on embryo production. Female (C57Bl/6×CBA) F1 hybrid mice were used for the experiment. Statistical significance. *corresponds to *p* < 0.01 according to the Mann–Whitney U test

We compared the productivity of female mice after hormonal stimulation with either an eCG–hCG mixture or a combination of these hormones injected simultaneously with the inhibin antiserum. In the former case, the median oocyte yield was nine zygotes; however, addition of anti-inhibin antibodies to eCG increased the number of ovulated oocytes obtained from each mouse by 275% (Mann–Whitney U test, *p* < 0.01) (median number, 34 oocytes; maximum, 50 oocytes) (*Fig. 2*, *Table*). This modification made it possible to reduce the number of animals required for the experiments, which also reduced the cost of breeding and housing and, most important, increased the yield of the embryos that had survived microinjection of the gene construct and were subsequently transferred to mouse foster mothers.

**Choosing the strain of mouse zygote donors**

A number of studies have demonstrated that the genetic background of a zygote donor mouse is important in order to ensure efficiency in the generation of genome- edited mice [26-28]. Female F1 hybrids derived from the genetically characterized and commonly used parental inbred C57Bl/6 (B6) strain are quite popular. Thanks to the effect of hybrid vigor, female F1 hybrid mice are known to have improved fertility, better respond to superovulation, and produce more oocytes [29, 30]. Zygotes obtained from these mice survive microinjection up to eight times more efficiently compared to inbred strains [16]. However, embryos obtained from F1 parents genetically differ from each other and carry various combinations of genetic polymorphisms that differ in their initial inbred strains, which may cause random errors because of the potential effect that differences in the genetic background could have on the phenotype [30]. In order to mitigate these unfavorable conditions, transgenic mice obtained through genome editing of a F1 hybrid need to be backcrossed with one of the inbred parental strains. This increases the cost of animal breeding and housing, as well as lengthens the time interval between the birth of genome-edited mice and experiment initiation.

**Fig. 3:**
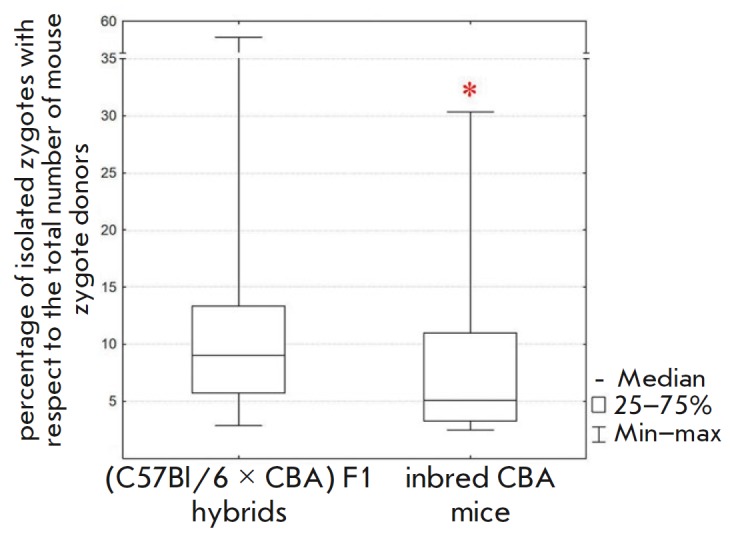
The influence of the genetic background of mice on embryo production. Female inbred CBA and (C57Bl/6 × CBA) F1 hybrid mice were compared. Superovulation was triggered by sequential administration of eCG and hCG. Statistical significance. *corresponds to *p* < 0.05 according to the Mann–Whitney U test

Inbred strains having identical genomes are preferred in experiments that address the phenotype of transgenic mice. The B6 strain is the most common genetic background of transgenic mice. Nevertheless, despite the good response of young female B6 mice to superovulation stimulants [30], their unicellular embryos have grained cytoplasm and small, poorly distinguishable pronuclei. Furthermore, zygotes in B6 mice poorly tolerate microinjection, which increases embryonic mortality in this strain [31, 32]; therefore. it seems inefficient to use it to generate transgenic mice.

**Fig. 4:**
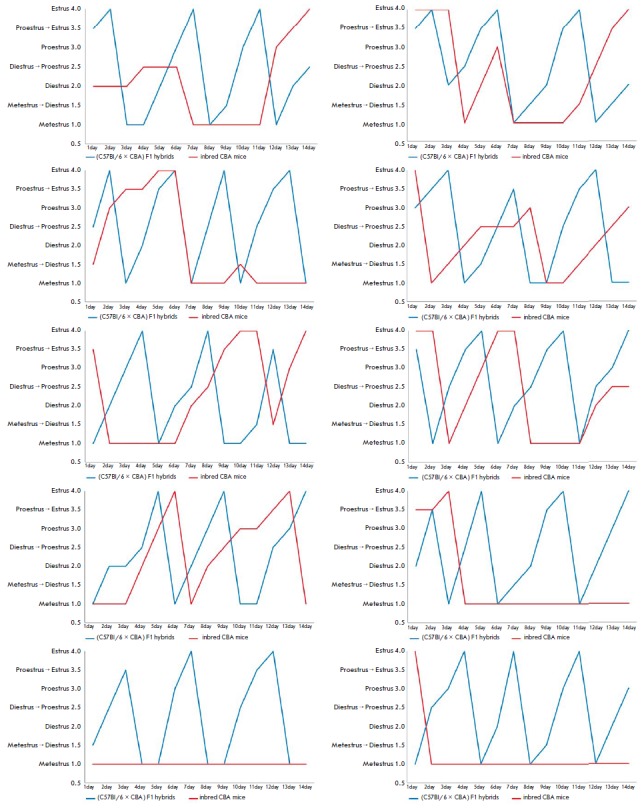
Estrous cycle dynamics in female inbred CBA and (C57Bl/6 × CBA) F1 hybrid mice

**Fig. 5:**
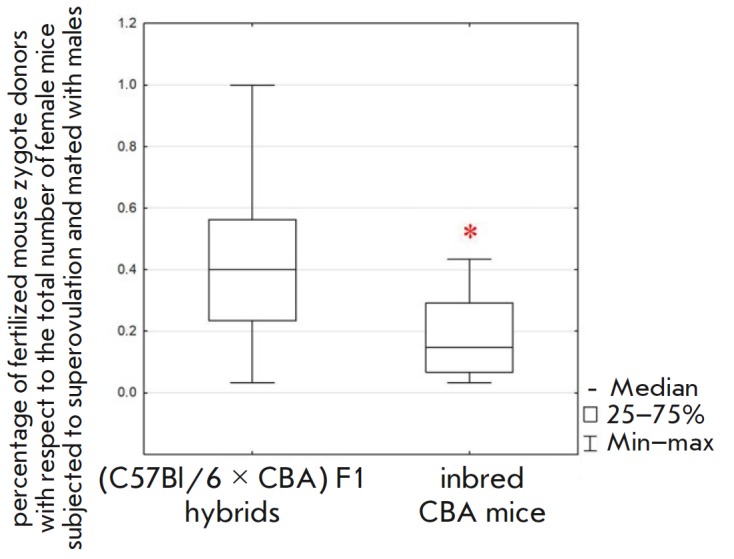
Breeding efficiency of female CBA and (C57Bl/6 × CBA) F1 hybrid mice. Statistical significance. *corresponds to *p* < 0.01 according to Mann–Whitney U test

We decided to assess the productivity of female mice of the CBA strain, which is commonly used as a parental strain whose crossing with C57Bl/6 yields F1 hybrids [16, 33]. At this stage, a combination of eCG and hCG (without the inhibin antiserum added) was used for the superovulation of the female CBA and F1 mice used as controls. It was established that inbred CBA mice produce fewer zygotes by 21% (Mann–Whitney U test, *p * < 0.05) compared to hybrid mice (*Fig. 3*, *Table*). This can be attributed to the different concentrations of endogenous hormones or different sensitivity of the ovaries to exogenous gonadotropins, which may affect the number of ovulated oocytes [34]. To elucidate the potential reasons for the low reproductive parameters of the CBA strain, we tested the estrous cycle regularity in this mouse strain. It turned out that compared to hybrid mice (in which estrus occurs every 4–5 days, which is normal for laboratory mice [8, 9, 35]), CBA mice were significantly (Mann–Whitney U test, *p* < 0.05) more likely to have delayed onset of estrus and prolongation of the metestrus phase (*Fig. 4*). Efficiency in mating was lower for female CBA mice by 55% (Mann–Whitney U test, *p* < 0.01) than it was for the hybrid mice (*Fig. 5*, *Table*). These data indicate that female CBA mice have an irregular estrous cycle, which is probably a factor responsible for the low mating efficiency and poor response to superovulation. Furthermore, according to published data, the embryos derived from female CBA mice are inferior to those of hybrids in a number of parameters; thus, they tolerate the microinjection procedure and cryoconservation much worse than embryos derived from hybrids [33].

Since the data presented above and our own findings show that the CBA and C57Bl/6 strains are ill-suited for the generation of a large number of zygotes [31, 32], we needed to choose an inbred strain that could be used for this purpose.

**Fig. 6:**
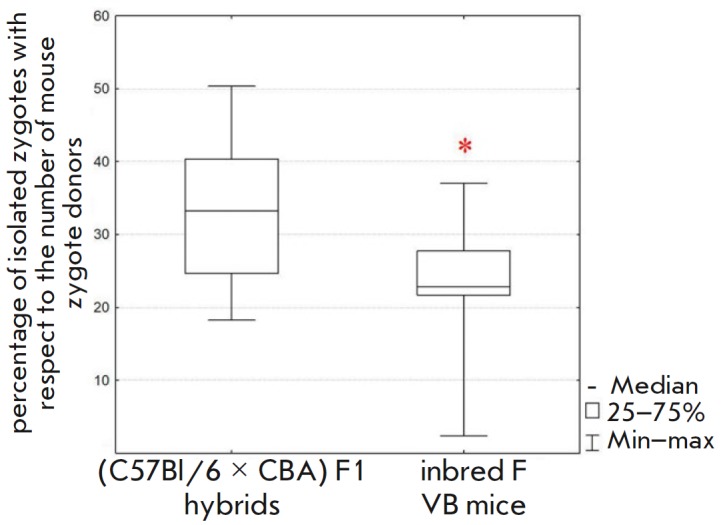
Influence of the genetic background of mice on embryo production. Females inbred FVB and (C57Bl/6 × CBA) F1 hybrid mice were compared. Superovulation was caused by sequential administration of eCG with inhibin antiserum and hCG. Statistical significance. *corresponds to the* p* < 0.05 according to the Mann–Whitney U test

Zygotes in the FVB strain were earlier reported to be suitable for pronuclear injections [30]. We tested whether this strain could be used by applying a modernized superovulation system containing the inhibin antiserum. We demonstrated that female FVB mice respond to superovulation less efficiently and produce 32% fewer zygotes (Mann–Whitney U test, *p* < 0.05) compared to F1 hybrids (*Fig. 6*, *Table*).

**Fig. 7:**
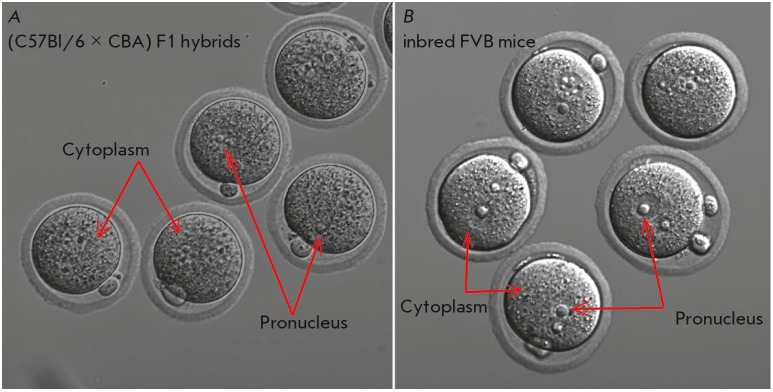
Features of the structure of a donor embryo intended for microinjection *A *– the granular cytoplasm and the poorly visible pronuclei of (C57Bl/6 × CBA) F1 hybrid mice. *B *– Homogeneous cytoplasm and clearly defined pronuclei of inbred FVB mice

**Fig. 8:**
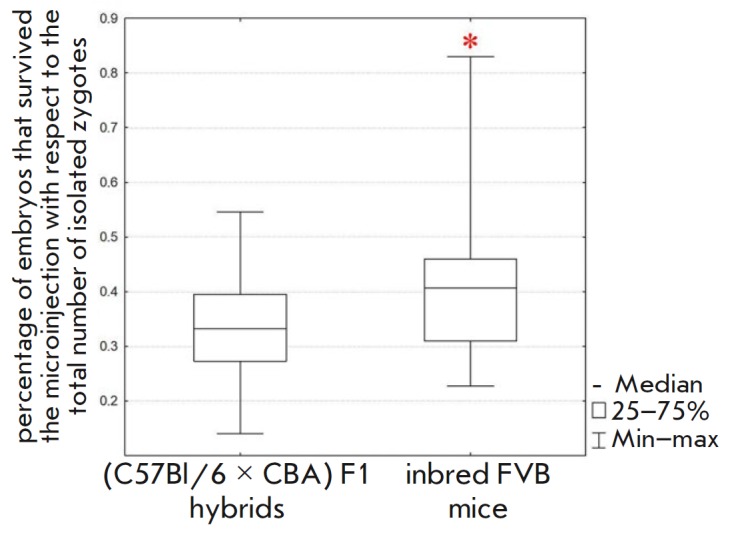
The influence of the genetic background of mice on embryo survival after microinjection. Female inbred FVB and (C57Bl/6 × CBA) F1 hybrid mice were compared. Statistical significance. *corresponds to the *p* < 0.05 according to the Mann–Whitney U test

Although the FVB mice produced a smaller median number of ovulated oocytes compared to that for the (B6 × CBA) F1 hybrids, unicellular embryos in the FVB strain had “pristine” nongrained cytoplasm and large, clearly defined pronuclei, which are good targets for microinjections (*Fig. 7*). This was a crucial factor for the successful genome-editing procedure. We also proved the earlier reported data [36, 32] that FVB embryos are highly resistant to microinjection. Our findings demonstrate that the survival rate of FVB embryos after microinjection is 22% higher (Mann–Whitney U test, *p* < 0.05) than that of F1 hybrids (*Fig. 8*, *Table*). Hence, we inferred that female inbred FVB mice are the best candidates for producing embryos that can be subsequently used for genome editing.

**Choosing the mouse strain to be used as foster mothers**

The next stage in the technology of generating transgenic mice involves the transfer of the microinjected embryos into the infundibulum and their intrauterine development. At this stage, the choice of the strain for producing pseudopregnant female mice (foster mothers) plays a crucial role [16, 19, 37]. The microinjection procedure is extremely traumatizing for the embryos. A comparison of the native and microinjected embryos showed that the latter exhibit a significant delay in embryonic development [38]. This fact places a special responsibility on the researcher who performs the surgery and chooses a mouse foster mother, since a number of limitations can be encountered when performing this task. Because of the small reproductive tract and positive pressure in the mouse oviduct, the embryos transferred to the infundibulum can be repulsed into the open ovarian cavity. Blood and/or mucus at the capillary tip can plug the capillary and cause embryo loss during the surgery. Finally, defects in uterine responsiveness and uterine contractions can also cause pregnancy failure [39].

In order to optimize the transfer of microinjected embryos and ensure a stable gestation course, as well as successful birth and survival of the litter, the strain of mice used as foster mothers needs to have good reproductive characteristics and marked maternal behavior [5]. This has been proved by the reports that the rates of embryo implantation and birth of mice having different genetic backgrounds largely depend on the genetic background of the mouse foster mothers [37].

It is inefficient to use inbred mice as foster mothers [40]. Most frequently, these mice are “bad” mothers, so the litter of genome-edited mouse pups can die. The death of most of the microinjected embryos after their transferred to the oviduct of a foster mother is a separate challenge. If only one or two embryos in the mother’s uterus survive, they can grow so large that they would not be able to be born without damaging themselves and/or the foster mother. Furthermore, female mice of some strains can be incapable of nursing small litters; so, the newborn transgenic mice will also die [5]. Female F1 hybrids are often chosen as foster mothers [18, 19, 28], as they are regarded as “good” mothers that can give birth to and preserve even litters consisting of two pups [19, 37]. It has been reported that both hybrid and outbred mice are used as foster mothers [32, 41, 42].

**Fig. 9:**
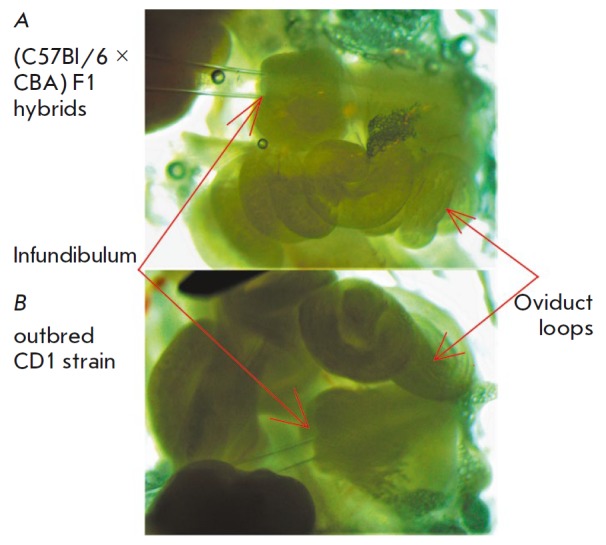
The features of the infundibulum structure in foster mice. *A *– in (C57Bl/6 × CBA) F1 hybrid mice, the infundibulum having thick walls and a narrow entrance. *B *– in female CD1 mice, the infundibulum is large and has thin walls and a wide entrance

In this study, we compared the efficiency of using female hybrid F1 (B6 x CBA) mice and outbred CD1 mice as foster mothers. A three-year study demonstrated that no significant intergroup differences in pregnancy efficiency and characteristics of material behavior exist between F1 and CD1 mice. Both of these strains can be successfully used as foster mothers. However, it is much more convenient to use female CD1 mice rather than hybrids for embryo transfer surgery. Indeed, according to the reports from Charles River Laboratories, a world leader in the commercial production of laboratory rodents, outbred CD1 mice are ideal candidates for surgery and as foster mothers [43]. A distinctive feature of female CD1 mice is that they have a larger oviduct ampulla compared to that in (B6 × CBA) F1 mice [44]. In our turn, we also found out that female CD1 mice have a large oviduct with thinner walls and a wide infundibulum compared to F1 hybrids (*Fig. 9*, *Table*). It is equally efficient to use female outbred CD1 mice and (B6 × CBA) F1 hybrids as pseudopregnant recipients in the generation of transgenic animals.

## CONCLUSIONS

Having analyzed different schemes of generation of transgenic mice, we conducted a series of studies to determine the experimental conditions that would be optimal for each protocol stage:

1. Superovulation using the inhibin antiserum significantly (almost threefold) increases the productivity of mouse zygote donors compared to the conventional superovulation procedure;

2. It is most reasonable to use FVB mice (whose zygotes have pronuclei with well-defined boundaries and whose embryos are characterized by a high survivability after microinjection) as mouse zygote donors. This mouse strain does not need to be backcrossed with the inbred parental strain;

3. Both female outbred CD1 mice and (B6 × CBA) F1 hybrids can be used as foster mothers.
